# A signature of 13 autophagy‑related gene pairs predicts prognosis in hepatocellular carcinoma

**DOI:** 10.1080/21655979.2021.1880084

**Published:** 2021-02-23

**Authors:** Jie Cao, Lili Wu, Xin Lei, Keqing Shi, Liang Shi

**Affiliations:** a Translational Medicine Laboratory, The First Affiliated Hospital of Wenzhou Medical University, Wenzhou, China; b Department of Clinical Transfusion, The Eighth Affiliated Hospital, Sun Yat-sen University, Shenzhen, China; c Department of Clinical Laboratory Medicine, The Eighth Affiliated Hospital, Sun Yat-sen University, Shenzhen, China

**Keywords:** Autophagy-related gene pairs, hepatocellular carcinoma, prognosis, immune

## Abstract

Growing evidences suggest that autophagy plays a momentous part in the tumorigenesis and development of hepatocellular carcinoma (HCC). However, there are not many researches to predict the prognosis of HCC using autophagy-related genes. Therefore, based on the clinical information and RNA-Seq data of The Cancer Genome Atlas data portal (TCGA), 13 autophagy‑related gene pairs were screened to build the autophagy‑related signature to predict the prognosis by least absolute shrinkage and selection operator (LASSO) regression analysis. Besides, the International Cancer Genome Consortium (ICGC) cohort was further applied to verify the autophagy‑related prognostic signature. Gene ontology (GO), Kyoto Encyclopedia of Genes and Genomes (KEGG) and gene set enrichment analysis (GSEA) were also used to predict the relevant function of the autophagy-related gene pairs signature. As shown in the results, the autophagy-related gene pairs were mainly involved in process utilizing autophagic mechanism, autophagy, macroautophagy and cellular response to oxidative stress. The immune cell levels in the high-risk and low-risk group were explored, which showed that the three immune cells were obviously increased in the high-risk group, while the five immune cells were obviously increased in the low-risk group. In conclusion, an autophagy‑related prognostic signature was established to predict the prognosis of HCC patients with great accuracy and we found that autophagy‑related prognostic signature was related to infiltrating immune cells.

## GRAPHICAL ABSTRACT

**Figure:**
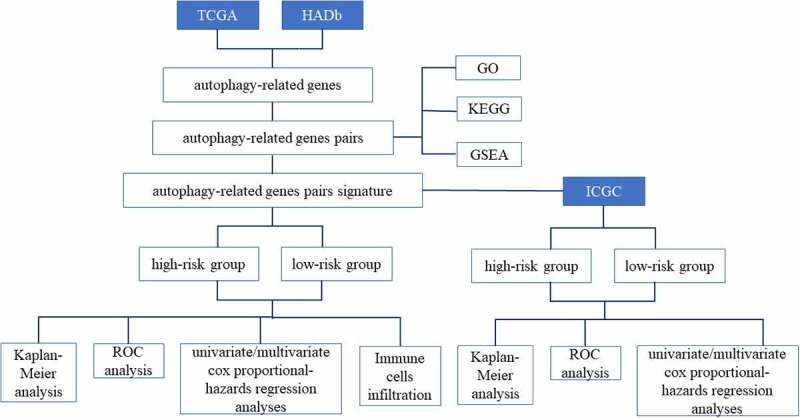

## Introduction

According to GLOBAOCAN, liver cancer was one of the most deadly cancers around the world in 2018 [1]. The most frequent category of primary malignant liver cancers was hepatocellular carcinoma (HCC) [2]. Because of the high mortality of HCC, researching effective and stable biomarkers of HCC was a significant task [3]. Nevertheless, the molecular pathological mechanisms of the development of HCC are still unclear. The accurate prediction of HCC prognosis could contribute to individual treatment and reduce waste of resources. At present, the assessments of prognosis mainly include the tumor stage and the degree of liver function damage [4]. These methods could not accurately reflect the prognosis of HCC. Hence, it is urgent to develop and validate a novel prognostic assessment system.

Many researches have proposed signatures based on gene expression for outcome stratification in HCC patients. 11 genes, including TNRC6B, VAV3 andRNF213, with a range of mutational prevalence from 1% to 3%, could predict the prognosis of HCC patients [5]. Jing-Xian Gu et al. have established a signature of six-lncRNA to predict the prognosis of HCC [6]. The 4-gene signature, which was strongly correlated with the age, grade, and T stage of HCC patients, acted as a candidate prognostic factor [7]. However, the above signatures have not been widely used in clinical applications because of the lack of abundant validation and overfitting on small development datasets [8]. The normalization of gene expression level was insufficient in previous studies, which posed a significant challenge of the data analysis with high heterogeneity among diverse datasets and sequencing platforms differences [9]. Recently, a novel way based on corresponding rank relative to the gene expression values has been put forward to conquer the defects of the gene expression levels normalization and scaling, and has obtained reliable consequences in many researches [10,11].

The word autophagy date from the Greek word, denoting ‘self-eating’, and refers to the collection of various processes that deliver cellular materials to lysosomes for digestion. In mammals, micro-autophagy, macro-autophagy [12], and chaperone-mediated autophagy [13] are currently known. Three main types of autophagy, of which macro-autophagy is the most studied form. Because the impact of autophagy was complex in cancer, many studies have looked at the relationship between autophagy and HCC. For example, ATG14, an autophagy-related protein, was abnormally increased in HCC, which is related with the poor prognosis of patients with HCC [14]. In addition, autophagy was connected with pattern recognition receptor (PRR), cell death pathways and inflammatory in tumor cells, and therefore changed the immunogenicity of the tumor microenvironment (TME) and the anti-tumor immune response development [15]. Nevertheless, the prognosis effect and the relationship with immune cells of autophagy have not been clarified in HCC.

In the study, we established the signature of autophagy-related gene pairs through analyzing the gene expression profile of HCC. the prognosis of HCC patients could be predicted using the signature. Moreover, the association of signature of autophagy-related gene pairs and tumor microenvironment was further analyzed.

## Methods

### Acquisition of HCC datasets and autophagy‑related genes

The RNA-seq expression matrix and corresponding clinic information of 374 HCC specimens were gained from The Cancer Genome Atlas (TCGA) database (https://portal.gdc.cancer.gov) [16], which was acted as the discovery dataset. The International Cancer Genome Consortium (ICGC) database (https://icgc.org/) including 243 samples was served as the validation dataset [17]. 232 autophagy-associated genes were further obtained from the Human Autophagy Database (HADb, http://autophagy.lu/clustering/index.html) [18]. The expression data of autophagy-associated genes were extracted from the discovery dataset and the validation dataset. Subsequently, the autophagy-related genes with uneven distribution (determined by median absolute deviation < 0.5) and relatively small change were removed [19]. Consequently, we obtained 127 autophagy-associated genes, which were shared between the two cohorts.

### Establishment of autophagy-related gene pairs signature

The score for each autophagy-related gene pair was computed by the paired comparison of the gene expression levels in each patient. Based on the suggested method, if the expression level of preceding gene was much higher than the latter gene expression level in a specific autophagy-related gene pair, the score of this autophagy-related gene pair was 1, otherwise, the score was 0. The common autophagy-related gene pairs in two cohorts were screened out as the candidate gene pairs to reflect the prognosis of HCC. The Kaplan-Meier analysis coupled with the log-rank test was applied for the univariate analysis, which was used to select autophagy-related gene pairs related with prognosis. The least absolute shrinkage and selection operator (LASSO) analysis was applied to establish the ﬁnal model [20]. The autophagy-related gene pairs signature was computed as following: Prognostic score = (Exprgenepair-1 × Coefgenepair-1) + (Exprgenepair-2 × Coefgenepair-2) + … + (Exprgenepair-n × Coefgenepair-n) [21]. Exprgenepair was the gene pair expression value and Coefgenepair was the LASSO regression coefficient. The cut off of the autophagy-related gene pairs signature was determined by time-dependent receiver operating characteristic (ROC) curve for the overall survival (OS) in the TCGA cohort [22].

### Validation of autophagy-related gene pairs signature validation

In order to validate the prognostic signature, the prognostic score was calculated by the autophagy-related gene pairs signature in the ICGC cohort. Furthermore, the HCC patients were also separated into high risk and low risk group based on the identical cut-off value in the ICGC cohort. The log-rank test was performed to compared survival curves between two groups. Meanwhile, patients with different tumor stage and grade were also performed Kaplan-Meier analysis. The ROC curve analysis was applied to demonstrate the specificity and sensitivity and of autophagy-relevant gene pairs signature in outcome prediction. Univariate/multivariate cox-regression models were applied to estimate risk elements that impacted OS.

### Functional analysis of autophagy-related gene pairs signature

To explore the potential functions of the autophagy-related gene pairs signature, GO analysis and KEGG analysis were performed by the ‘clusterProfiler’ package. Biological processes were collected from the Gene Set Enrichment Analysis (MSigDB C2 and C5 databases) (https://www.gsea-msigdb.org/gsea/index.jsp) [23]. The false discovery rate (FDR) adjusted p-value below 0.05 was considered signiﬁcant.

### Profiling of immune cells infiltration

CIBERSORT algorithm, a novel computational method, uses 547 reference gene expression values for estimating enrichment of various immunocyte compositions [24]. In our research, CIBERSORT was performed to detect the abundance of 22 infiltrating immune cells in the high risk and low risk group. According to the Monte-carlo sampled, the deconvolution p-values of all patients were computed to offer reliability in the assessment. The expression profiling of gene of TCGA was put on the online platform CIBERSORT website (http://cibersort.stanford.edu/) for analysis with the default signature matrix at 1000 permutations.

### The analysis of correlation

The heat map of correlation was performed by the ‘corplot’ package, which was aimed to explore the correlation of autophagy-related gene pairs. The relationships between infiltrating immune cells and risk score of patients were also performed.

### Statistical analysis

The statistical analyses were processed by R version 3.6.0 software (https://www.r-proje ct.org/) and Perl version 5.32.0.1 software (https://www.perl.org/). The differences among the two groups were compared with student’s t-tests. The ‘survival’ package was used to perform the survival analysis. The ROC curves were performed by ‘survivalROC’ package. For all studies, P-value < 0.05 was served as statistically significantly different. Statistical signiﬁcance was displayed as *P < 0.05, **P < 0.01, ***P < 0.001.

## Results

In this study, we aimed to establish autophagy‑related gene pairs signature for predicting the prognosis in patients with HCC. We filtered out autophagy‑related gene to make the gene pairs and these autophagy‑related gene pairs were applied to construct a prognostic signature. Function enrichment analysis was applied to analyze the potential functions of autophagy‑related gene pairs. In addition, according to the autophagy‑related gene pairs signature, we further explored the infiltration levels of 22 immune cells in the high-risk and low-risk group to explore the correlation between and immune cells and autophagy‑related gene pairs signature.

### Construction of the autophagy-related gene pairs signature

**Figure 1.**
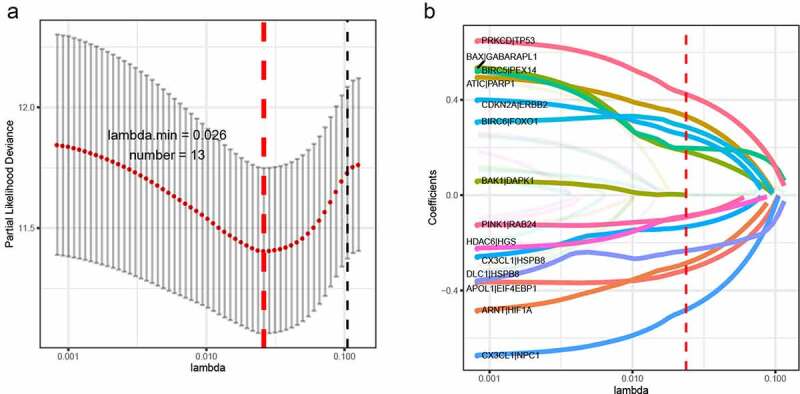
LASSO cox-regression analysis to filter the autophagy-related gene pairs for prognosis. (a)The most representative autophagy-related gene pairs were obtained by LASSO analysis; (b) LASSO coefficient of the 13 autophagy-related gene pairs

**Figure 2.**
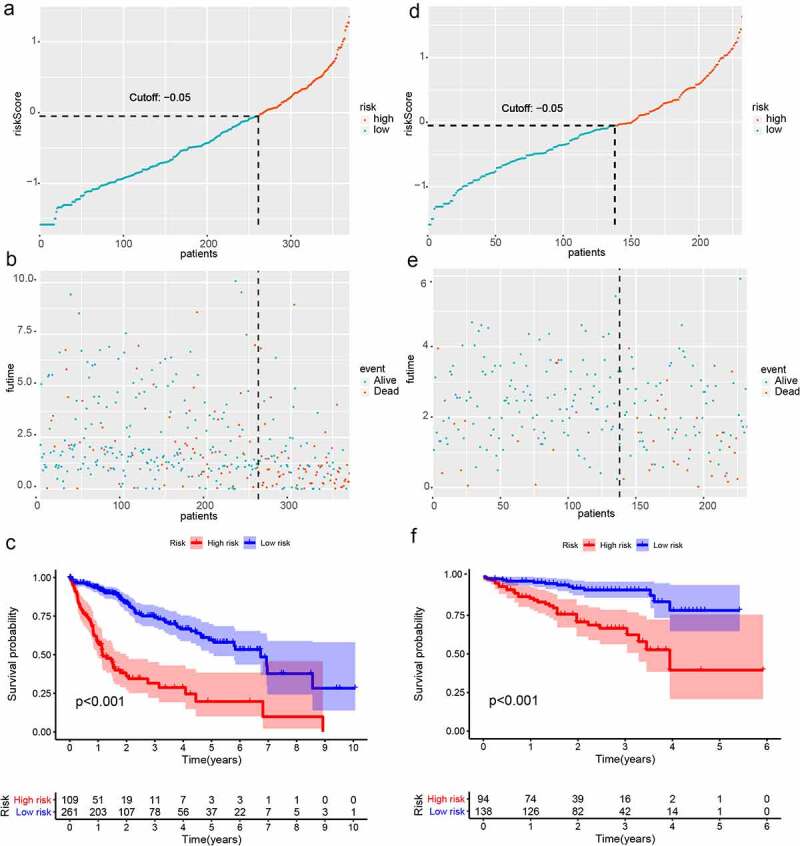
Survival analysis of HCC in the two cohort. (a)Risk scores distribution of patients in the training cohort; (b) Survival time and survival state of patients in the training cohort; (c) Kaplan-Meier survival curve of patients with HCC in the training cohort; (d) Risk scores distribution of patients with HCC in the validation cohort; (e) Survival time and survival state of patients with HCC in the validation cohort; (f) Kaplan-Meier analysis of patients in the validation cohort

**Figure 3.**
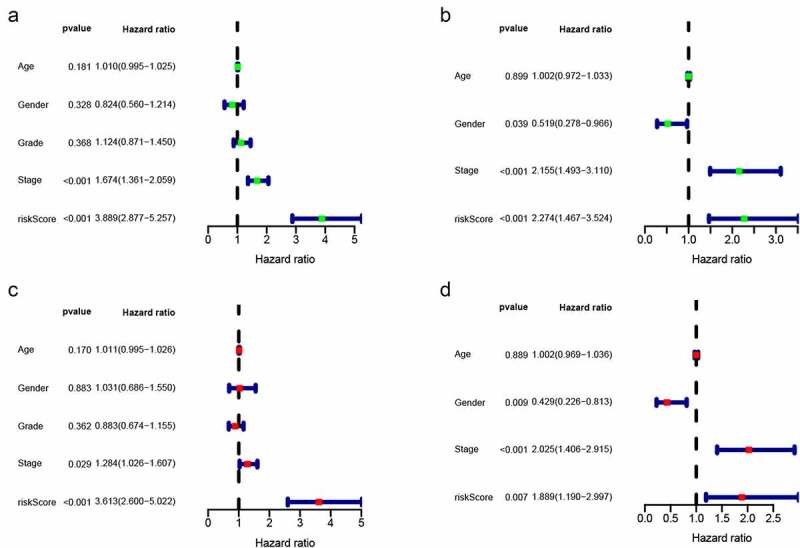
Univariate and multivariate analyses showed independently prognostic factors for OS of HCC in the two cohorts. (a) Univariate analyses in the training cohort; (b) Multivariate analyses in the training cohort; (c) Univariate analyses in the validation cohort; (d) Multivariate analyses in the validation cohort

The TCGA dataset gene expression profiling was used as a training cohort. According to the median-absolute-deviation (MAD) >0.5, genes with more large variation were proposed as candidate genes. Among 232 autophagy-relevant genes from HADb, 127 autophagy-related genes were obtained by two cohorts, which were applied to construct 1454 autophagy-related gene pairs (Figure S1A, Table 1, Table S1). The univariate Cox-regression analysis was applied to filter the autophagy-related gene pairs, which were associated with OS in the TCGA cohort. Hence, we found that 32 autophagy-related gene pairs were obviously related to OS in HCC patients. Subsequently, we built the signature consisting of 13 gene pairs by lasso analysis in the training group (Figure 1). The autophagy-related gene pairs signature included a panel of 24 unique autophagy-related genes. The correlation analysis of 13 autophagy-related gene pairs showed that most of the gene pairs had a significant relationship (Figure S2A): BIRC5| PEX14 with BAX|GABARAPL1, CX3CL1|HSPB8 with DCL1|HSPB8 and BIRC5| PEX14 with PINK1|RAB24 especially (Figure S2B-2D). Then we calculated all HCC patient risk scores in the training group according to the autophagy-related gene pairs signature (Figure 2a). Based on the ROC analysis at 1 year, the patients were assigned into high-risk group or low-risk group by optimal cut-off value with a set at −0.05 (Figure S3A). Our study indicated that the low-risk group had a significantly better survival outcome than the high-risk group (P < 0.001; HR = 3.7 (2.8, 4.9)) (Figure 2b,Figure 2c). In order to further study the prognostic ability of the clinic information and this signature, univariate/multivariate cox proportional-hazards regression analyses were performed in the training group. The univariate Cox analysis displayed that clinical information like tumor stage and autophagy-associated gene pairs signature could affect the prognosis (Figure 3a). Nevertheless, multivariate Cox analysis indicated that autophagy-associated gene pairs signature remained an independent prognosis factor (Figure 3c). The ROC analysis was applied to estimate the signature, which indicated that the AUC for 1, 3, 5 year survival times were 0.817, 0.774, and 0.765, respectively (Figure S3H).

**Table 1.** 127 autophagy-related genes

127 autophagy-related genes					
APOL1	ARNT	ARSA	ARSB	ATF4	ATF6
ATG4B	ATG4D	ATIC	BAG1	BAG3	BAK1
BAX	BCL2L1	BECN1	BID	BIRC5	BIRC6
BNIP3	BNIP3L	CANX	CAPN1	CAPN2	CAPNS1
CASP1	CASP3	CASP4	CASP8	CCL2	CD46
CDKN1A	CDKN1B	CDKN2A	CHMP2B	CHMP4B	CLN3
CTSB	CTSD	CX3CL1	CXCR4	DAPK1	DDIT3
DLC1	DNAJB1	DNAJB9	DRAM1	EDEM1	EEF2
EEF2K	EGFR	EIF2AK2	EIF4EBP1	ERBB2	ERN1
FAS	FKBP1A	FKBP1B	FOS	FOXO1	FOXO3
GAA	GABARAPL1	GAPDH	GOPC	HDAC1	HDAC6
HGS	HIF1A	HSP90AB1	HSPA5	HSPA8	HSPB8
IKBKE	ITGA3	ITGA6	ITGB1	ITGB4	KIF5B
KLHL24	LAMP1	LAMP2	MAP1LC3A	MAP1LC3B	MAPK1
MAPK3	MAPK8IP1	MTOR	MYC	NAMPT	NBR1
NCKAP1	NFE2L2	NFKB1	NPC1	P4HB	PARP1
PEA15	PELP1	PEX14	PEX3	PIK3R4	PINK1
PPP1R15A	PRKAR1A	PRKCD	PTK6	RAB24	RAB33B
RB1	RB1CC1	RGS19	SERPINA1	SESN2	SIRT1
SPHK1	SQSTM1	TMEM74	TNFSF10	TP53	TP53INP2
TSC2	TUSC1	ULK1	ULK2	VAMP3	VEGFA
WIPI1

### Validation and assessment of the autophagy-related gene pairs signature

The risk scores of all HCC patients were calculated by the above signature in the ICGC dataset. Subsequently, based on the cut-off value of −0.05, all patients were assigned into two groups: low-risk and high-risk and survival rate were also calculated. Consistent with the result acquired from the TCGA dataset, the low-risk patients had the better outcome than the high-risk patients (P < 0.001; HR = 2.3 (1.5, 3.5)) (Figure 2d-Figure 2f). In addition, we validated prognostic effect of the autophagy-relevant gene pairs signature in diverse tumor stage and grade. The result showed that the autophagy-related gene pairs signature could significantly predict survival outcome in stage I (P < 0.001; HR = 3.7 (2.2, 6.1)), stage II (P < 0.001; HR = 4.3 (2.2, 8.5)), stage 2162 (P < 0.001; HR = 3.2 (2.0, 5.4)), grade I (P < 0.001; HR = 4.6 (2.2, 9.7)), grade II (P < 0.001; HR = 3.3 (2.2, 5.1)) and grade III (P < 0.001; HR = 4.1 (2.5, 6.7))(Figure S3B-S3G).In the ICGC dataset, univariate Cox analysis indicated that the clinical information including gender and tumor stage showed a prognostic ability and multivariate Cox analysis demonstrated that the autophagy-related gene pairs signature remained an independent prognosis factor (Figure 3b,Figure 3d).

### Functional assessment of the autophagy-related gene pairs signature

**Figure 4.**
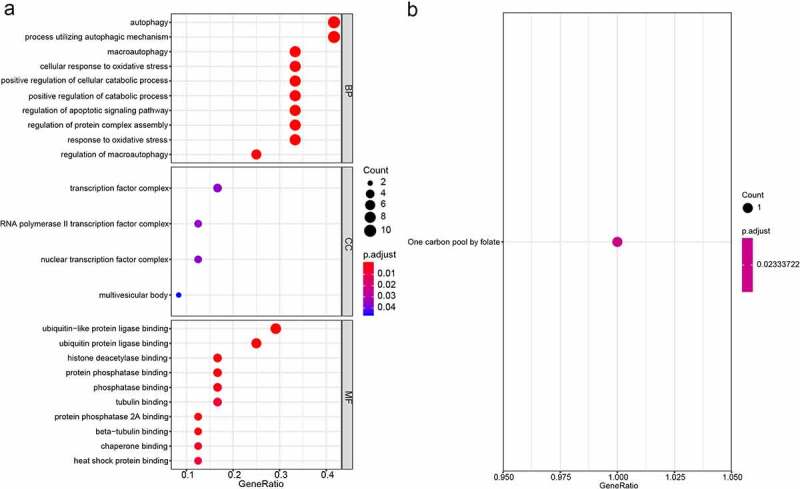
GO and KEGG enrichment analyses of the signature gene. (a) GO enrichment analyses; (b) KEGG enrichment analyses

To assess the potential function of the autophagy-related gene pairs signature, we performed the GO, KEGG, and GSEA analysis. The GO analysis consisting of biological processes (BP), molecular functions (MF) and cellular components (CC), and suggested that the autophagy-related gene pairs signature genes were mostly enriched in autophagy, transcription factor complex and ubiquitin-like protein ligase binding (Figure 4a). The KEGG enrichment analyses showed that signature genes only enriched in One carbon pool by folate (Figure 4b). GSEA analysis was carried out to analyze the pathways which were obviously altered between the high-risk group and low-risk group. ‘POSITIVE_REGULATION_OF_CELL_CYCLE’, ‘POSITIVE_REGULATION_OF_RESPONSE_TO_DNA_DAMAGE_STIMULUS’, ‘MITOTIC_DNA_INTEGRITY_CHECKPOINT’ and ‘MEIOTIC_CELL_CYCLE_PROCESS’ were obviously enriched in the high-risk groups (Figure S4).

### The immune cells infiltration between two groups

**Figure 5.**
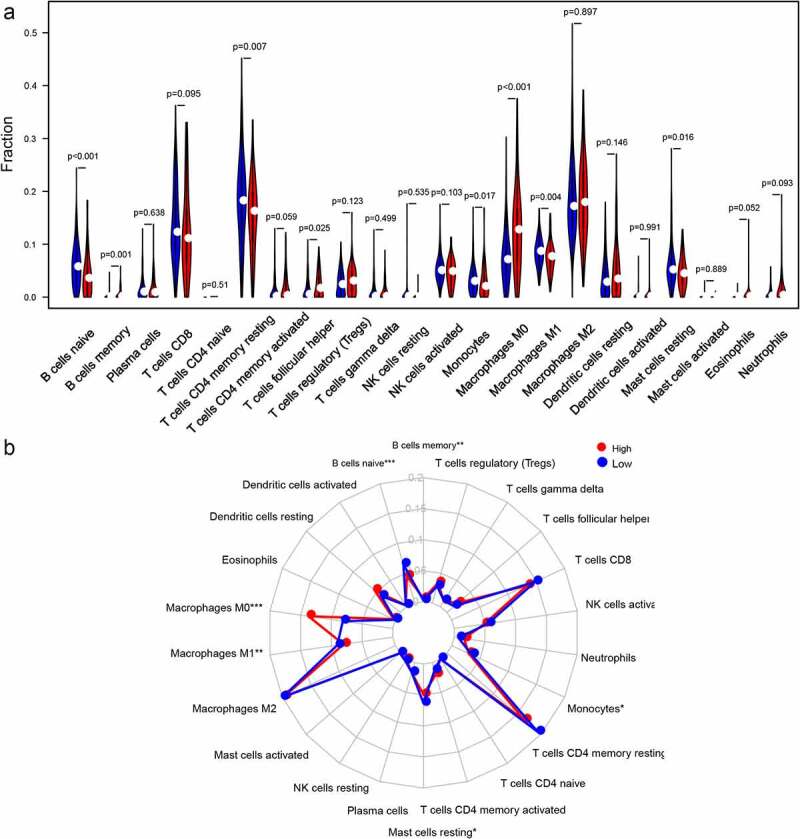
Immune infiltration levels in the two groups. (a)The radar plot displayed 22 different immune cell levels for different groups; (b) The violin plot displayed 22 different immune cell levels for different groups

According to the CIBERSORT, we estimated the relative proportion of the 22 immune cells for each HCC patient within the high-risk class and the low-risk class. The comparison of different immune cells in the low-risk class and high-risk class using CIBERSORT was displayed in Figure 5. B cells memory, T cells follicular helper and Macrophages M0 were obviously increased in the high-risk class than the low-risk class; however, the expression levels of B cells naive, Macrophages M1, T cells CD4 memory resting, Monocytes and Mast cells resting were obviously lower in the high-risk class (Figure. S5). Moreover, the potential correlations between the autophagy-related gene pairs signature score and the eight immune cells were calculated. Our results demonstrated that the autophagy-related gene pairs signature was remarkably positively correlated to the T cells follicular helper, B cells memory and Macrophages M0, and negatively correlated to the B cells naive, Macrophages M1, Mast cells resting and T cells CD4 memory resting. However, the autophagy-related gene pairs signature score had no association with the Monocytes (Figure. S6).

## Discussion

Despite of significant advances in the prevention, diagnosis, and therapy of HCC, it remained the primary reason of cancer death around the world [25–27]. According to exome sequencing, genomic characterization studies and transcriptome analysis, HCC had various molecular characteristics and clinical outcomes [28,29]. Because of the heterogeneity of cancer, patients of HCC should select different therapeutic approaches to achieve more significant therapeutic effects. Autophagy played different roles in different stages of HCC and markedly affected the efficacy of multiple therapeutic methods [30]. The analysis of autophagy-related proteins and pathways in HCC could be helpful for finding biomarkers of specific treatments and predicting the prognosis more accurately. In HCC, the molecular mechanism and clinical significance of autophagy remain unclear.

In this research, we obtained a novel prognosis signature from 13 autophagy-related gene pairs of HCC in TCGA cohort and verified in ICGC cohort. The autophagy signature could effectively separate patients into different subgroups according to their survival outcomes. Based on multivariate analysis and Kaplan-Meier method, autophagy-related gene pairs signature could act as the independent predictive factor of HCC patients. The above results were successfully verified in the ICGC cohort. In a word, autophagy-related genes may be closely associated with prognosis of HCC patients.

The traditional prognosis signatures required to preprocess the gene expression levels, which was the vital reason to restrain the wide use of models. The autophagy-related gene pairs signature was obtained through the relative sequencing of the gene expression levels, and paired comparison of weights was only carried out within the patient gene expression profiles, which was a huge advantage. Our prognosis signature could not only eliminate batch effects among different platforms but also did not need the normalization and scaling of data. The formula for the risk score and truncation value could be used in various datasets, which made it easier to be applied in clinical. This method has been performed robustly in cancer-relevant studies [31], and it was the primary superiority in our research.

Autophagy was associated with a wide range of physiological processes and human diseases, and it has been linked to both tumorigenesis and oncotherapy in recent years. Hence, it was important to build the autophagy-related signature for predict the survival outcome for HCC patients. Our 13 autophagy-related gene pairs signature contained 26 genes, which were mainly participated in the functions of autophagy and tumor occurrence and development. For example, the Tp53, as the gene of autophagy-related prognosis signature, could contribute to the chemoresistance and cell survival in HCC under conditions of nutritional deficiency by modulating the activation of autophagy [32]. In addition, the study has demonstrated that HDAC6 could regulate the tumor progression by suppressing the signaling pathway of canonical Wnt/β-catenin in HCC [33].

More and more evidences have shown that the tumor microenvironment (TME) was intricately related to cancer initiation, promotion, and progression. And autophagy and immunity took great participation in TME. Some studies have demonstrated that autophagy played a momentous role in the immunity regulation. Autophagy regulated cellular differentiation, development and homeostasis of multiple immune cells like monocytes, T-cells, and B-cells, thus was deeply involved in the innate immune system as well as lymphocyte activation and survival [34]. For instance, the Bcl-2/Bax ratio was significantly associated with both the absolute number and the rate of CD4(+) T cells and natural killer cell activity in head and neck cancer patients [35]. CX3CL1, served as the autophagy-related gene, was significant associated with NK cell-mediated cytotoxicity, T cell activation, and leukocyte migration in lung adenocarcinoma, and a study demonstrated that the differential expression of CX3CL1 could affect the migration activity of CD8(+) T cells and CD56(+) NK cells in hepatoma cells, which had hepatitis B virus replication [36]. All of the above-mentioned autophagy-related genes, the changes of immune cells, and related pathways and cytokines stated clearly that autophagy, immunity and cancer were all linked.

Nevertheless, there are limitations in this research. Firstly, we adopted a retrospective method to analyze the data, and to validate the results, a prospective cohort needs to be done. Secondly, more details about the biological functions and molecular mechanisms of 24 autophagy-related genes in HCC patients required further assessment. RT-PCR could be used for further clinical applications.

## Conclusion

In summary, we established the autophagy-related gene pairs signature with data from the TCGA, ICGC and HADb datasets, which could accurately predict the HCC patient prognosis. The results demonstrated that the low-risk group had a significantly better OS than the high-risk. Moreover, we discovered that the immune cell infiltration levels were obviously different between high-risk group and low-risk group, which indicated that the autophagy-related gene pairs signature was relevant to infiltrated immune cells. The autophagy-related gene pairs signature will act as a crucially predictive marker to offer insights into the therapeutic strategies.

## Supplementary Material

Supplemental Material

## Data Availability

The datasets generated and/or analyzed during the current study are available in TCGA (https://portal.gdc.cancer.gov) and ICGC (https://icgc.org/).

## Highlights

1. Signature of 13 autophagy‑related gene pairs could predict the HCC prognosis.
2. Autophagy-related gene pairs were involved in autophagy and oxidative stress.
3. The immune cell levels in the high-risk and low-risk group were different.
