# Supplementary material for: A signature of 13 autophagy‑related gene pairs predicts prognosis in hepatocellular carcinoma
Source: Bioengineered. 2021 Feb 23;12(1):697–707. doi: 10.1080/21655979.2021.1880084 (PMC8806227; doi:10.1080/21655979.2021.1880084)
Supplement: Supplemental Material [file KBIE_A_1880084_SM5215.zip › Supplementary information/Table S1.docx]

| **Table S1. The full name of 127 autophagy-related genes** | |
| --- | --- |
| **Symbol** | **Full Name** |
| APOL1 | apolipoprotein L1 |
| ARNT | aryl hydrocarbon receptor nuclear translocator |
| ARSA | arylsulfatase A |
| ARSB | arylsulfatase B |
| ATF4 | activating transcription factor 4 |
| ATF6 | activating transcription factor 6 |
| ATG4B | autophagy related 4B cysteine peptidase |
| ATG4D | autophagy related 4D cysteine peptidase |
| ATIC | 5-aminoimidazole-4-carboxamide ribonucleotide formyltransferase/IMP cyclohydrolase |
| BAG1 | BAG cochaperone 1 |
| BAG3 | BAG cochaperone 3 |
| BAK1 | BCL2 antagonist/killer 1 |
| BAX | BCL2 associated X, apoptosis regulator |
| BCL2L1 | BCL2 like 1 |
| BECN1 | beclin 1 |
| BID | BH3 interacting domain death agonist |
| BIRC5 | baculoviral IAP repeat containing 5 |
| BIRC6 | baculoviral IAP repeat containing 6 |
| BNIP3 | BCL2 interacting protein 3 |
| BNIP3L | BCL2 interacting protein 3 like |
| CANX | calnexin |
| CAPN1 | calpain 1 |
| CAPN2 | calpain 2 |
| CAPNS1 | calpain small subunit 1 |
| CASP1 | caspase 1 |
| CASP3 | caspase 3 |
| CASP4 | caspase 4 |
| CASP8 | caspase 8 |
| CCL2 | C-C motif chemokine ligand 2 |
| CD46 | CD46 molecule |
| CDKN1A | cyclin dependent kinase inhibitor 1A |
| CDKN1B | cyclin dependent kinase inhibitor 1B |
| CDKN2A | cyclin dependent kinase inhibitor 2A |
| CHMP2B | charged multivesicular body protein 2B |
| CHMP4B | charged multivesicular body protein 4B |
| CLN3 | CLN3 lysosomal/endosomal transmembrane protein, battenin |
| CTSB | cathepsin B |
| CTSD | cathepsin D |
| CX3CL1 | C-X3-C motif chemokine ligand 1 |
| CXCR4 | C-X-C motif chemokine receptor 4 |
| DAPK1 | death associated protein kinase 1 |
| DDIT3 | DNA damage inducible transcript 3 |
| DLC1 | DLC1 Rho GTPase activating protein |
| DNAJB1 | DnaJ heat shock protein family (Hsp40) member B1 |
| DNAJB9 | DnaJ heat shock protein family (Hsp40) member B9 |
| DRAM1 | DNA damage regulated autophagy modulator 1 |
| EDEM1 | ER degradation enhancing alpha-mannosidase like protein 1 |
| EEF2 | eukaryotic translation elongation factor 2 |
| EEF2K | eukaryotic elongation factor 2 kinase |
| EGFR | epidermal growth factor receptor |
| EIF2AK2 | eukaryotic translation initiation factor 2 alpha kinase 2 |
| EIF4EBP1 | eukaryotic translation initiation factor 4E binding protein 1 |
| ERBB2 | erb-b2 receptor tyrosine kinase 2 |
| ERN1 | endoplasmic reticulum to nucleus signaling 1 |
| FAS | Fas cell surface death receptor |
| FKBP1A | FKBP prolyl isomerase 1A |
| FKBP1B | FKBP prolyl isomerase 1B |
| FOS | Fos proto-oncogene, AP-1 transcription factor subunit |
| FOXO1 | forkhead box O1 |
| FOXO3 | forkhead box O3 |
| GAA | alpha glucosidase |
| GABARAPL1 | GABA type A receptor associated protein like 1 |
| GAPDH | glyceraldehyde-3-phosphate dehydrogenase |
| GOPC | golgi associated PDZ and coiled-coil motif containing |
| HDAC1 | histone deacetylase 1 |
| HDAC6 | histone deacetylase 6 |
| HGS | hepatocyte growth factor-regulated tyrosine kinase substrate |
| HIF1A | hypoxia inducible factor 1 subunit alpha |
| HSP90AB1 | heat shock protein 90 alpha family class B member 1 |
| HSPA5 | heat shock protein family A (Hsp70) member 5 |
| HSPA8 | heat shock protein family A (Hsp70) member 8 |
| HSPB8 | heat shock protein family B (small) member 8 |
| IKBKE | inhibitor of nuclear factor kappa B kinase subunit epsilon |
| ITGA3 | integrin subunit alpha 3 |
| ITGA6 | integrin subunit alpha 6 |
| ITGB1 | integrin subunit beta 1 |
| ITGB4 | integrin subunit beta 4 |
| KIF5B | kinesin family member 5B |
| KLHL24 | kelch like family member 24 |
| LAMP1 | lysosomal associated membrane protein 1 |
| LAMP2 | lysosomal associated membrane protein 2 |
| MAP1LC3A | microtubule associated protein 1 light chain 3 alpha |
| MAP1LC3B | microtubule associated protein 1 light chain 3 beta |
| MAPK1 | mitogen-activated protein kinase 1 |
| MAPK3 | mitogen-activated protein kinase 3 |
| MAPK8IP1 | mitogen-activated protein kinase 8 interacting protein 1 |
| MTOR | mechanistic target of rapamycin kinase |
| MYC | MYC proto-oncogene, bHLH transcription factor |
| NAMPT | nicotinamide phosphoribosyltransferase |
| NBR1 | NBR1 autophagy cargo receptor |
| NCKAP1 | NCK associated protein 1 |
| NFE2L2 | nuclear factor, erythroid 2 like 2 |
| NFKB1 | nuclear factor kappa B subunit 1 |
| NPC1 | NPC intracellular cholesterol transporter 1 |
| P4HB | prolyl 4-hydroxylase subunit beta |
| PARP1 | poly (ADP-ribose) polymerase 1 |
| PEA15 | proliferation and apoptosis adaptor protein 15 |
| PELP1 | proline, glutamate and leucine rich protein 1 |
| PEX14 | peroxisomal biogenesis factor 14 |
| PEX3 | peroxisomal biogenesis factor 3 |
| PIK3R4 | phosphoinositide-3-kinase regulatory subunit 4 |
| PINK1 | PTEN induced kinase 1 |
| PPP1R15A | protein phosphatase 1 regulatory subunit 15A |
| PRKAR1A | protein kinase cAMP-dependent type I regulatory subunit alpha |
| PRKCD | protein kinase C delta |
| PTK6 | protein tyrosine kinase 6 |
| RAB24 | RAB24, member RAS oncogene family |
| RAB33B | RAB33B, member RAS oncogene family |
| RB1 | RB transcriptional corepressor 1 |
| RB1CC1 | RB1 inducible coiled-coil 1 |
| RGS19 | regulator of G protein signaling 19 |
| SERPINA1 | serpin family A member 1 |
| SESN2 | sestrin 2 |
| SIRT1 | sirtuin 1 |
| SPHK1 | sphingosine kinase 1 |
| SQSTM1 | sequestosome 1 |
| TMEM74 | transmembrane protein 74 |
| TNFSF10 | TNF superfamily member 10 |
| TP53 | tumor protein p53 |
| TP53INP2 | tumor protein p53 inducible nuclear protein 2 |
| TSC2 | TSC complex subunit 2 |
| TUSC1 | tumor suppressor candidate 1 |
| ULK1 | unc-51 like autophagy activating kinase 1 |
| ULK2 | unc-51 like autophagy activating kinase 2 |
| VAMP3 | vesicle associated membrane protein 3 |
| VEGFA | vascular endothelial growth factor A |
| WIPI1 | WD repeat domain, phosphoinositide interacting 1 |
