# Supplementary figures and images for: A signature of 13 autophagy‑related gene pairs predicts prognosis in hepatocellular carcinoma
Source: Bioengineered. 2021 Feb 23;12(1):697–707. doi: 10.1080/21655979.2021.1880084 (PMC8806227; doi:10.1080/21655979.2021.1880084)

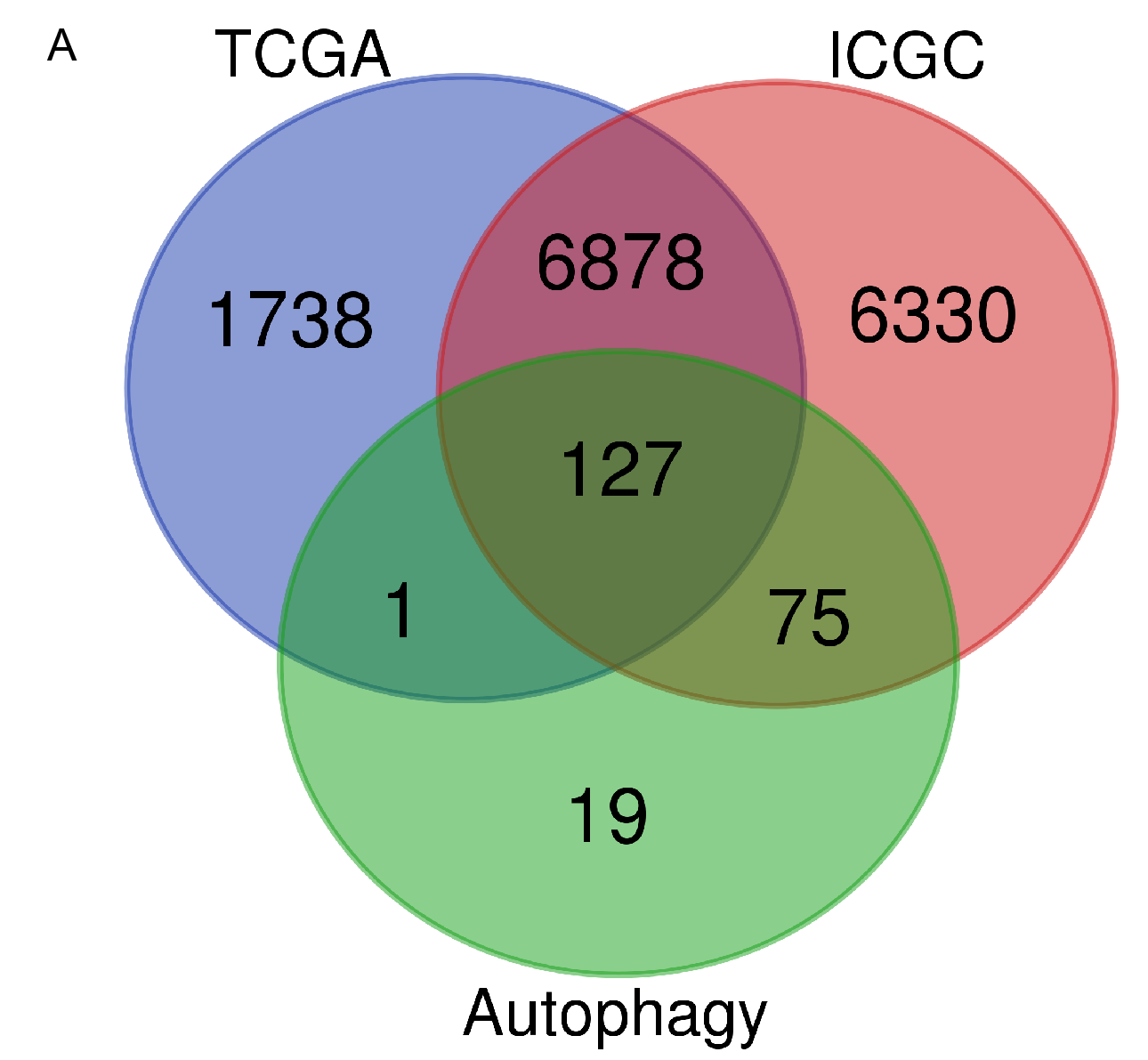

Supplement: Supplemental Material [file KBIE_A_1880084_SM5215.zip › Supplementary information/Figure S1.tif]

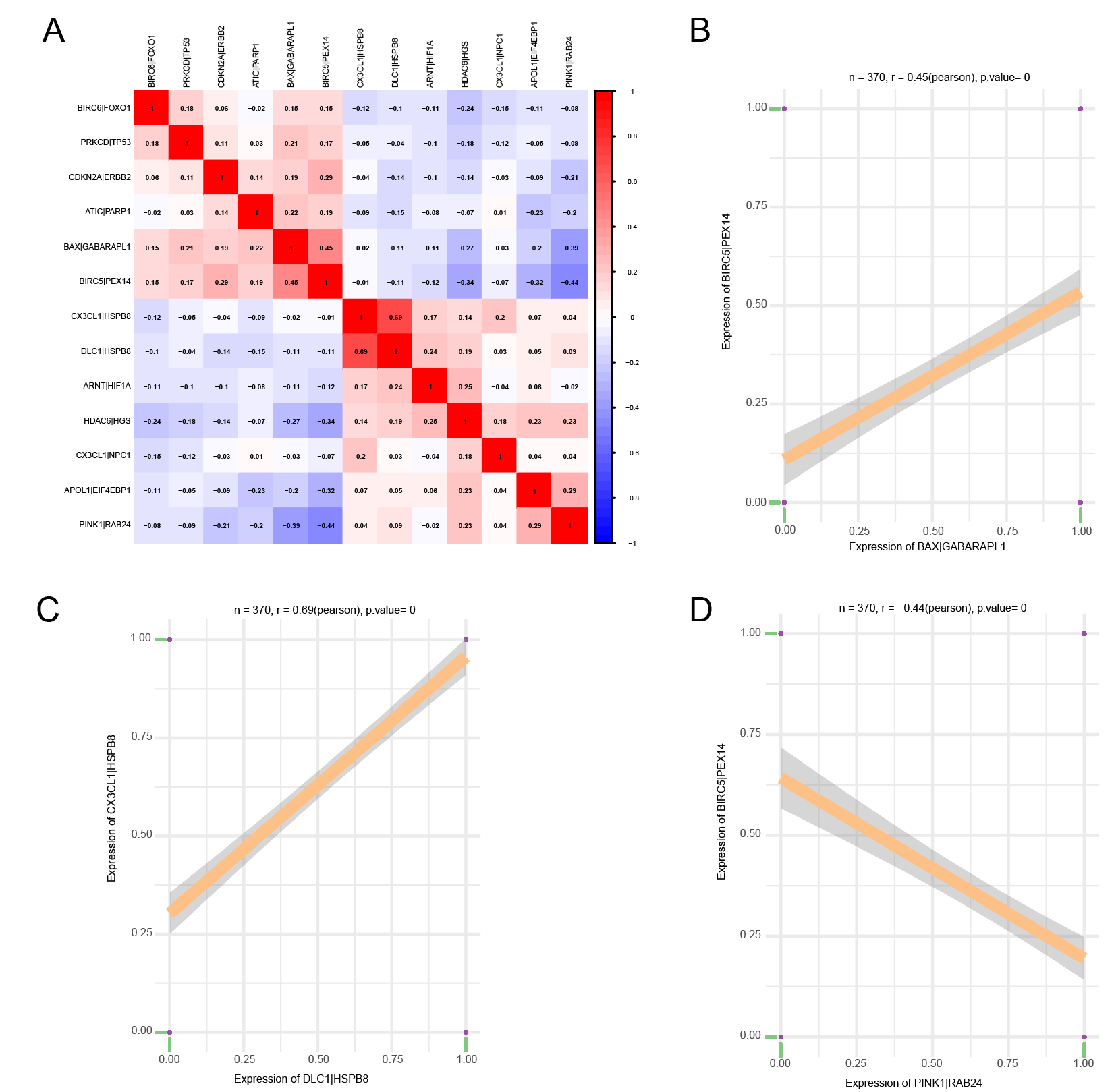

Supplement: Supplemental Material [file KBIE_A_1880084_SM5215.zip › Supplementary information/Figure S2.tif]

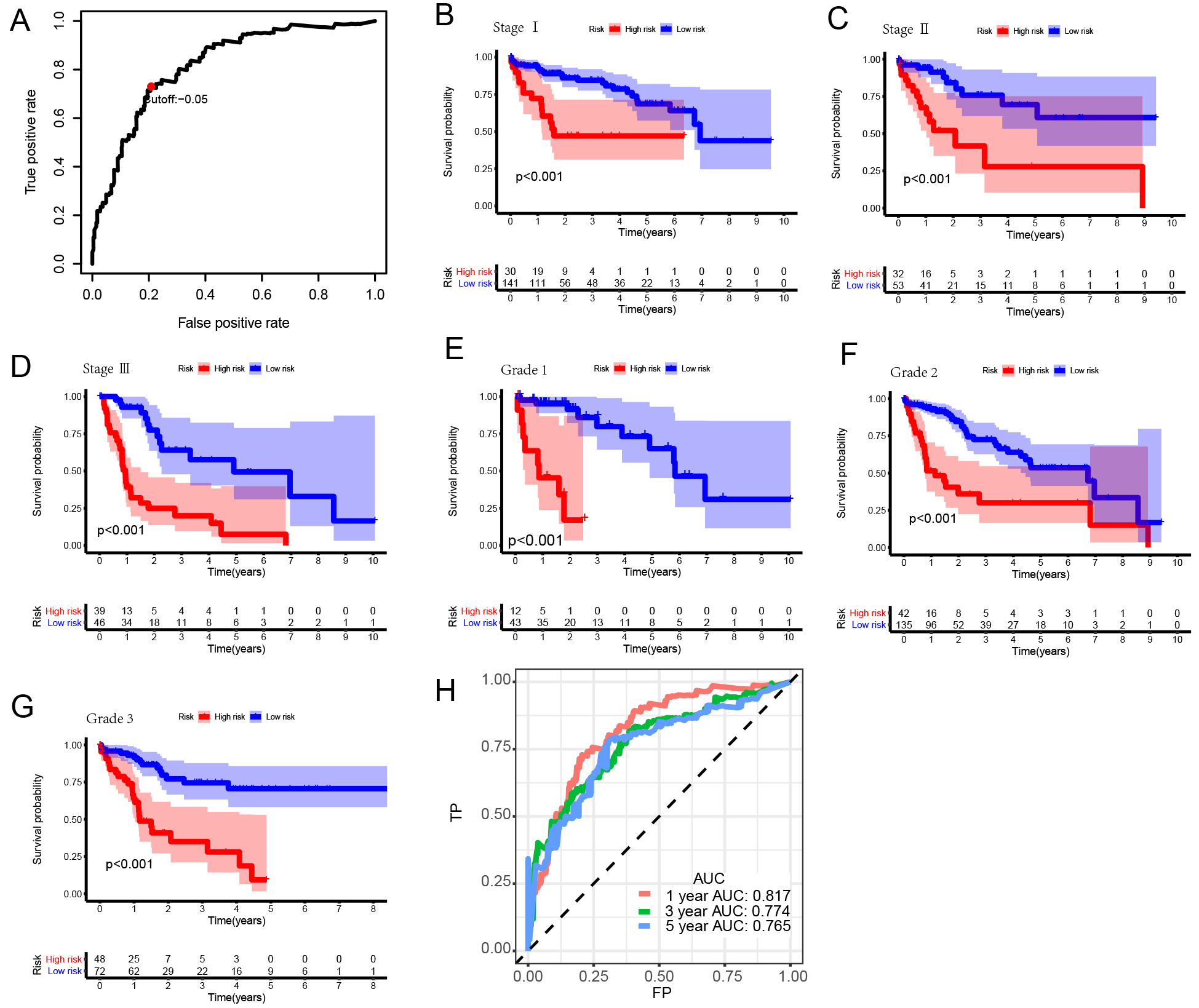

Supplement: Supplemental Material [file KBIE_A_1880084_SM5215.zip › Supplementary information/Figure S3.tif]

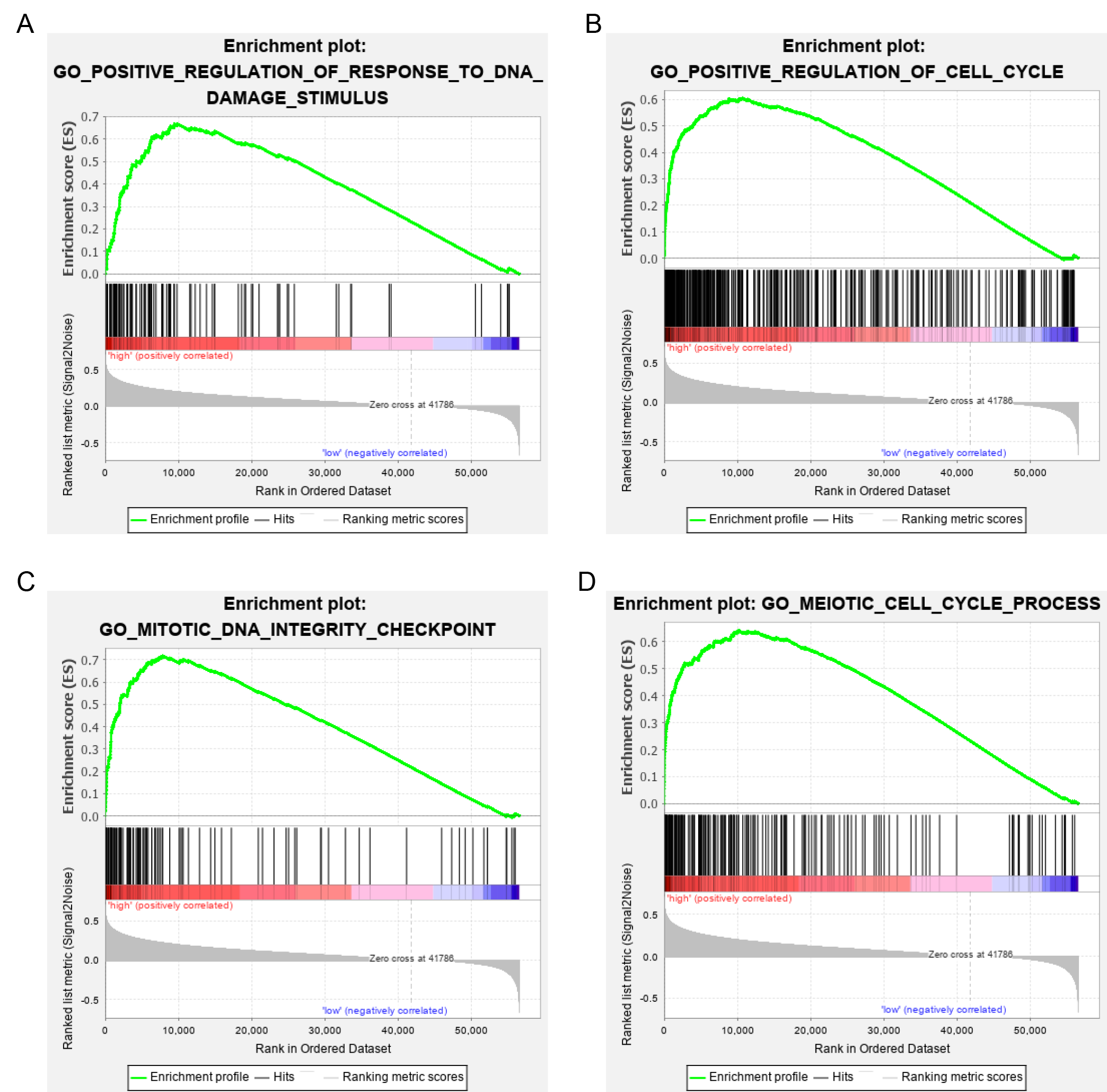

Supplement: Supplemental Material [file KBIE_A_1880084_SM5215.zip › Supplementary information/Figure S4 (1).tif]

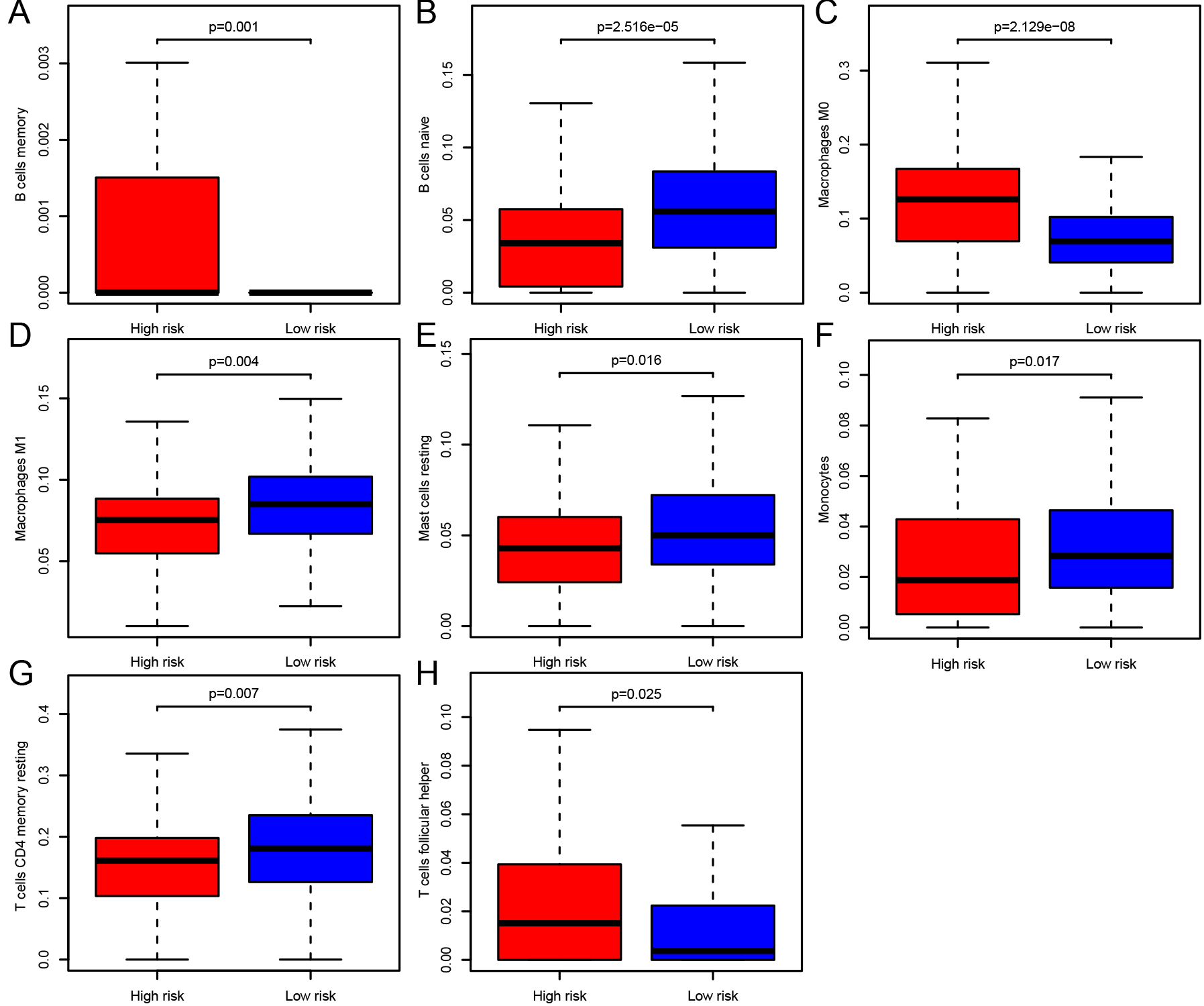

Supplement: Supplemental Material [file KBIE_A_1880084_SM5215.zip › Supplementary information/Figure S5.tif]

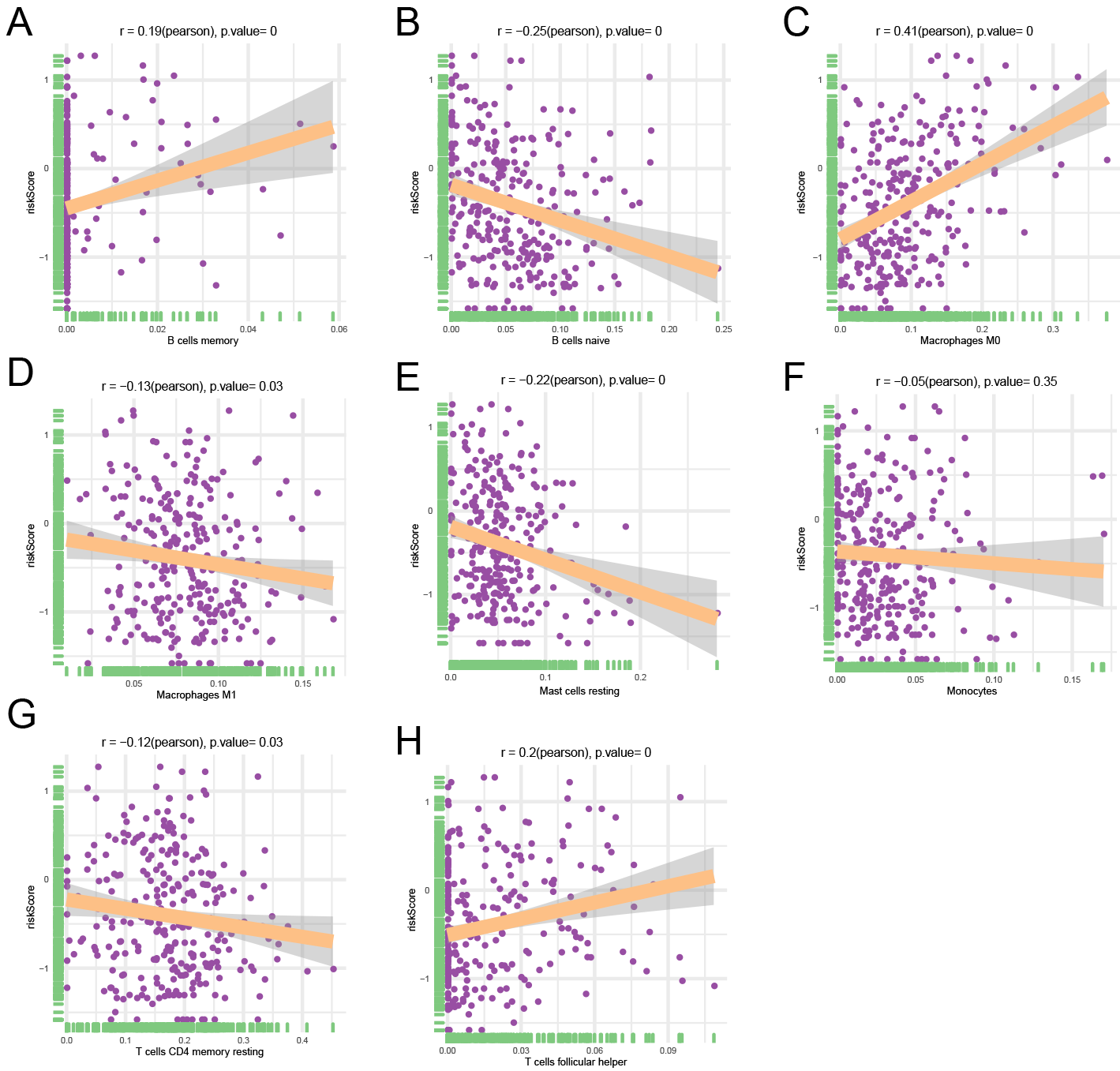

Supplement: Supplemental Material [file KBIE_A_1880084_SM5215.zip › Supplementary information/Figure S6.tif]

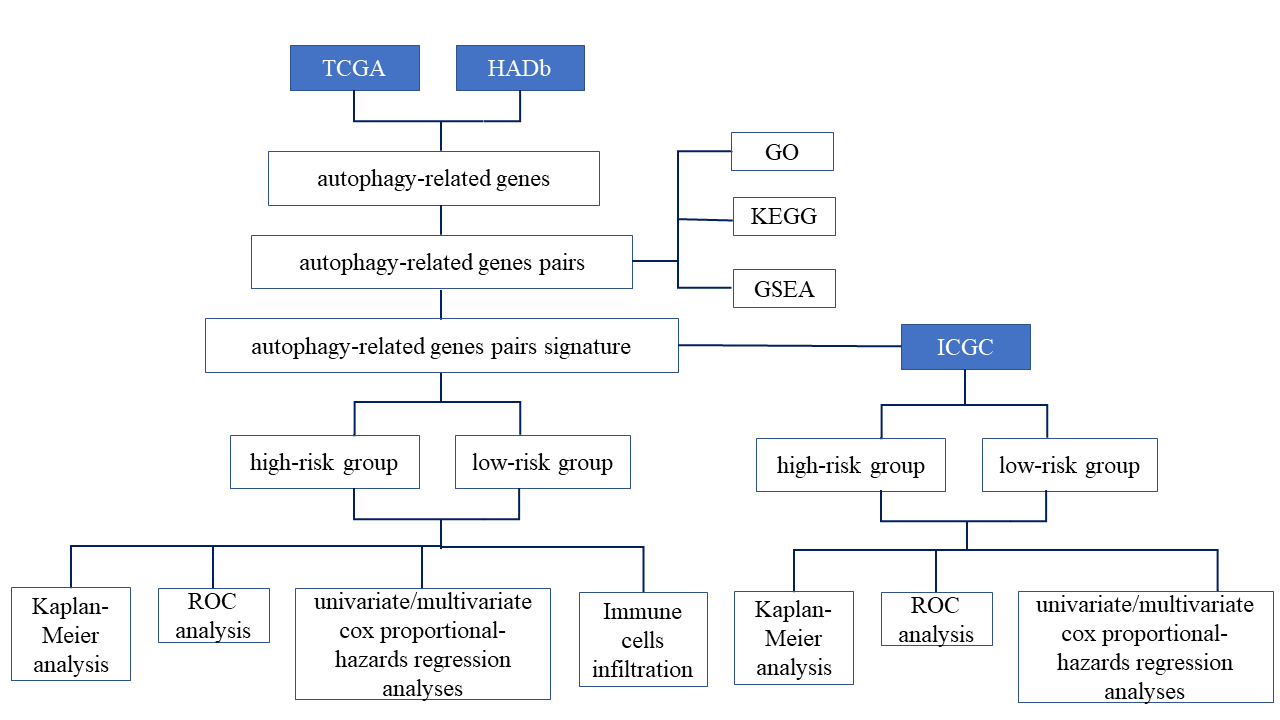

Supplement: Supplemental Material [file KBIE_A_1880084_SM5215.zip › Supplementary information/Graphical Abstract (1).tif]
